# Computer Simulations Suggest a Key Role of Membranous Nanodomains in Biliary Lipid Secretion

**DOI:** 10.1371/journal.pcbi.1004033

**Published:** 2015-02-18

**Authors:** Johannes Eckstein, Nikolaus Berndt, Hermann-Georg Holzhütter

**Affiliations:** Charité—University Medicine Berlin, Institute of Biochemistry, Berlin, Germany; University of California San Diego, UNITED STATES

## Abstract

The bile fluid contains various lipids that are secreted at the canalicular membrane of hepatocytes. As the secretion mechanism is still a matter of debate and a direct experimental observation of the secretion process is not possible so far, we used a mathematical model to simulate the extraction of the major bile lipids cholesterol, phosphatidylcholine and sphingomyelin from the outer leaflet of the canalicular membrane. Lipid diffusion was modeled as random movement on a triangular lattice governed by next-neighbor interaction energies. Phase separation in liquid-ordered and liquid-disordered domains was modeled by assigning two alternative ordering states to each lipid species and minimization of next-neighbor ordering energies. Parameterization of the model was performed such that experimentally determined diffusion rates and phases in ternary lipid mixtures of model membranes were correctly recapitulated. The model describes the spontaneous formation of nanodomains in the external leaflet of the canalicular membrane in a time window between 0.1 ms to 10 ms at varying lipid proportions. The extraction of lipid patches from the bile salt soluble nanodomain into the bile reproduced observed biliary phospholipid compositions for a physiologi-cal membrane composition. Comparing the outcome of model simulations with available experi-mental observations clearly favors the extraction of tiny membrane patches composed of about 100–400 lipids as the likely mechanism of biliary lipid secretion.

## Author Summary

Formation of the bile is one of the central functions of the liver. The bile fluid aids in the digestion of edible fats and removal of drugs and toxins from the body. The bile fluid is mainly composed of bile salts (BS), phosphatidylcholine (PC) and cholesterol (CH) in a fairly fixed proportion that prevents liver impairment by gallstone formation or cholestasis. During bile formation, BS are actively pumped out of the hepatocyte into the extracellular space where they extract PC and CH from the canalicular membrane. This extraction process bears the risk for the canalicular membrane to be destructed. Hence, only a certain fraction of the membrane should be accessible to the solubilizing activity of BS. We have developed a mathematical model that describes the temporal formation of CH-enriched ordered and PC-enriched disordered nanodomains in the canalicular membrane. Model simulations reveal that the disordered nanodomains exhibit a composition of PC and CH similar to that also found in the bile. From this finding and the good concordance of model simulations with experimental data we conclude that PC and CH are mainly secreted into the bile from the disordered nanodomain. Our work adds a new layer of physiological importance to the spontaneous formation of lipid domains in biological membranes.

## Introduction

One central function of the liver is the production of the bile which is indispensable for the efficient digestion of dietary lipids, elimination of hydrophobic xenobiotics and removal of cholesterol from the body. The bile is formed in the biliary canaliculi, i.e. the extracellular space that faces the canalicular membrane of hepatocytes. About 80% of the bile content is bile salts (BS), while the other components are phospholipids (≈ 15%) and cholesterol (≈ 5%). The secretion rate and composition of the bile are important factors in metabolic regulation, malfunctioning leading to hepatocyte damage, hypercholesterolemia, advanced levels of high-density lipoproteins or formation of atherosclerotic plaques [1].

Different models have been proposed to account for the canalicular secretion of hepatocyte-born lipids into the bile [2–6]. One possible mechanism of lipid secretion consists in the BS driven extraction of single lipids into the lumen of the canaliculus followed by assembly of these lipids and BS in mixed-micelles (‘single-lipid extraction’). An alternative model developed on the basis of ultrastructural investigations suggests that lipid vesicles highly enriched in phosphatidylcholine are formed via BS facilitated exo-vesiculation of microdomains present in the exoplasmic hemi-leaflet of the canalicular membrane [7, 8]. These BS extractable microdomains appear to coexist with sphingolipid-enriched microdomains which cannot be solubilized by BS and thus represent potential localized target areas for the clustering of proteins involved in bile formation as, for example, ABC transporters, aquaporins and P-type ATPases [9, 10]. One may hypothesize a mechanism of lipid secretion where both BS extractable and BS non-extractable microdomains of different lipid composition coexist in the exoplasmic leaflet of the canalicular membrane. The first ones represent membrane patches that can be easily intercalated by BS, protruded into the canalicular space and finally be released, the latter ones containing most of the proteins required to enrich the outer leaflet with specific lipids like phosphatidylcholine and conferring stability of the membrane against detergents. In both models the solubilization of membrane lipids is mediated by BS that are actively pumped into the canalicular lumen. They rely on the coexistence of BS soluble and BS insoluble membrane areas as the canalicular membrane has to meet two opposing conditions: (*i*) Maintenance of integrity in the presence of high concentrations of BS which are powerful detergents and (*ii*) at the same time allowing the secretion of membrane lipids.

The formation of microdomains goes along with the segregation into liquid ordered and liquid disordered phases. The formation of different lipid phases is largely determined by the saturation profile of the fatty acid tails of the phospholipids and of sphingomyelin [11]. The tight interaction between cholesterol and sphingomyelin side chains usually representing long and saturated fatty acids allows for the formation of liquid ordered phases [12, 13], whereas the high content of unsaturated fatty acids of phospholipids usually leads to a lower interaction between these membrane lipids and thus entails a liquid-disordered fraction of the membrane. Phosphatidylcholine species found in the rat liver canalicular membrane and bile have a fairly hydrophilic fatty acid composition with palmitate (16:0) in sn1-position and an unsaturated fatty acid (mostly 18:1, 18:2, 20:4) in the sn2-position [14].

Most findings on microdomains have been obtained in *in vitro* experiments with lipid mixtures [15, 16] and giant unilamellar vesicles [17]. Depending on the number, type and relative abundance of lipids used to constitute these artificial membrane-like systems, one may observe tiny unstable nanodomains (≈ 10 nm) [18–20] or much larger (> 200 nm) and stable microdomains with life-times of several seconds containing up to 100,000 lipids. The problem is that microdomains of this size and life-time have never been observed in the membranes of living cells. Instead, the existence of much smaller (≈ 10 nm) and very short-lived (< 0.1 ms) microdomains has been inferred from donor-quenching FRET analysis [21, 22], atomic-force microscopy [23] or deuterium-based nuclear magnetic resonance [24]. Possible reasons for this remarkable instability of microdomains in membranes of living cells are thermodynamic variations and mechanical perturbations exerted by cell-cell and cell-matrix interactions. In order to distinguish these tiny and very unstable microdomains from their counterparts present in model membranes we will refer to them later on as nanodomains [11].

As the processes of nanodomain formation and lipid transfer from the external leaflet of the canalicular membrane into the lumen of the bile canaliculus cannot be directly monitored by experimental means we have developed a mathematical model to simulate the formation of nanodomains in the canalicular membrane and the extraction of the major bile lipids cholesterol (CH), phosphatidylcholine (PC) and sphingomyelin (SM) from BS soluble nanodomains. The model enables dynamic simulations of the transit from an initially random to a highly structured lipid distribution on a time scale of several milliseconds. With the model we address the problems of membrane self-organization, biliary phospholipid secretion and composition as well as membrane integrity.

## Material and Methods

### Mathematical Model: Structure, Calibration and Validation

We simulated the lipid dynamics by using a Potts model approach [25]. The membrane is represented by a triangular lattice with periodic boundary conditions. Each lattice site is occupied by exactly one lipid. The model comprises the membrane lipids CH, PC and SM which represent the prevailing membrane lipids in the canalicular membrane of hepatocytes. Note that the model variable PC actually represents also other phospholipids of the exoplasmic leaflet (e.g. ≈ 11% phosphatidylethanolamine) as well as different fatty acid tail compositions. This is a necessary simplification of the model as experimental information on the impact of glycerophospholipids other than PC on domain formation and lipid diffusion is not available.

We introduced next-neighbor interaction energies *w* for each pair of membrane lipids. Furthermore, similar as in [26] we assumed that each lipid may be either in a low or high ordered internal state. In the low-ordered state the bulky conformation of the fatty acid chains allows high flexibility and thus rapid movement while in the high-ordered state the fatty acid chains are arranged in a way that the hydrophobic interactions with adjacent lipids are strong and thus restricting lipid mobility. We model the property of lipids to cooperatively synchronize their ordering states by assigning a coupling strength $j({\text{X}}_{i}^{\sigma },{\text{X}}_{k}^{\sigma })$ between the ordering states *σ* of the lipid species X resident in neighboring lattice sites *i* and *k*. The ordering states ho and lo are represented by *σ* = −1 and *σ* = +1 respectively. The coupling $j({\text{X}}_{i}^{\sigma },{\text{X}}_{k}^{\sigma })$ of lipid pairs contributes to the total ordering energy of the membrane with a positive sign if the two neighboring lipids possess identical ordering states and with negative sign if the ordering states are different:

$$J=-\sum_{i=1}^{N}\sum_{k\in n(i)}j({\text{X}}_{i}^{\sigma },{\text{X}}_{k}^{\sigma })\sigma_{i}\sigma_{k}.$$

The first sum runs over all lattice sites *N* and the second over the 6 neighbors of lattice site *i*.

Self-organization of lipid domains comprising lipids that are predominantly present in the lo or ho state is achieved by minimizing this total ordering energy.

Our approach to enforce a phase separation of lipids is similar to the well-known *Ising* model of ferromagnetism in statistical mechanics describing the formation of phases with different atomic spin states (–1, +1) across a 2-dimensional lattice [27]. Furthermore, we introduce next-neighbor interaction energies for each pair of membrane lipids and describe the dynamics of membrane lipids as a process driven by minimization of the total interaction energy of the lattice. As the lipids may occur in two different ordering states, we assign different interaction energies, *w*_ho_(X_*i*_, X_*k*_) and *w*_lo_(X_*i*_, X_*k*_), to adjacent lipids of species X at lattice site *i* and *k* depending on whether these two lipids are both in the ho state or lo state, respectively. The total interaction energy of the membrane is

$$W=\sum_{i=1}^{N}\sum_{k\in n(i)}w_{\sigma }({\text{X}}_{i},{\text{X}}_{k}).$$

In cases where the ordering states *σ* at sites *i* and *k* are different we assign to this lipid pair the mean of the two interaction energies. Movement of lipids on the lattice is restricted to their pair-wise interchange of next neighbors. The dynamics of membrane lipids and the distribution of their mobility states are governed by the minimization of the total energy *E* which is a linear combination of the ordering energy *J* and the interaction energy *W*:

$$E=W+\gamma \cdot J=\sum_{i=1}^{N}\sum_{k\in n(i)}\left(w_{\sigma }({\text{X}}_{i},{\text{X}}_{k})+\gamma \cdot j({\text{X}}_{i}^{\sigma },{\text{X}}_{k}^{\sigma })\sigma_{i}\sigma_{k}\right).$$

The scaling factor *γ* relates the ordering energy *J* to the interaction energy *W*.

The values of *w_σ_*(X_*i*_, X_*k*_) and $j({\text{X}}_{i}^{\sigma },{\text{X}}_{k}^{\sigma })$ represent Gibb’s free energies which arise from a multitude of electrostatic, Van der Waals and hydrophobic interactions between head groups and fatty acid tails of neighboring membrane lipids [28]. In our thermodynamic-based approach the behavior of the lattice in the equilibrium state is fully determined by the changes of the free energy associated with either switching of neighbored lipids or changing the ordering state of lipids.

For the stochastic simulations of lipid movement we applied the Gillespie algorithm [29]. The core of this algorithm consists in assigning to each possible elementary process *p_ij_* of lipid switch between *i* and *j*, a rate *r(i, j)* that depends upon the local interaction energies at positions *i* and *j* where the process *p_ij_* is executed: *r*(*i*, *j*) = exp(*β*(*w_i_* +*w_j_*)). Here, *w_i_* = Σ_*k∈n(i*)_ *w_σ_*(X_*i*_, X_*k*_) is the interaction energy of a lipid of species X_*i*_ at site *i* with all its neighbors with species X_*k*_ and *β* = 1/*k*_B_ *T* the inverse temperature. The probability *P*(Δ*t*) that during the time span Δ*t* no elementary process occurs is related to the elementary rates by *P*(Δ*t*) = exp(–*r*_tot_Δ*t*), where *r*_tot_ = Σ_*Pij*_ *r*(*i*, *j*) is the total rate, defined as the sum of the elementary rates. This relation is used to randomly generate elementary time steps, Δ*t* = -ln *η/r*_tot_, where *η* are equally distributed random numbers between 0 and 1. After having randomly chosen the time step for the next elementary process to occur, the elementary process to be executed has to be specified. This is done by randomly choosing an elementary process, i.e. lipid switch, with its corresponding probability.

So far we neglected the process of changes in the ordering state. From the molecular-dynamics point of view, a change in the conformation of the fatty acid tail should occur much more frequently compared with a switch of neighbored lipids. As a consequence, the elementary process “change the ordering state of a selected lipid” occurs much more frequently than the elementary process “switch adjacent lipids”. Hence, it is reasonable to assume that a large number of changes in the ordering states of lipids will occur between two subsequent lipid switches so that at any time point the conformational energy *J* becomes minimal.

The rate with which a membrane lipid flips between two alternative ordering states is not known. Considering that such a flip requires only a change in the conformation of the fatty acid tails it is reasonable to assume that flips of the ordering state occur with much higher rates than changes in the spatial position of the membrane lipid.

We thus refrained from executing explicitly the elementary process “change the ordering state of a selected lipid”. Instead, we make the steady-state (or equivalently: partial fast-equilibrium) assumption that the ordering energy is minimal and thus the ordering states of the lipids are in equilibrium at any time point. This also alleviates us from knowing the exact value of the scaling factor *γ*, as it now does not occur in the algorithm itself.

**Figure 1:**
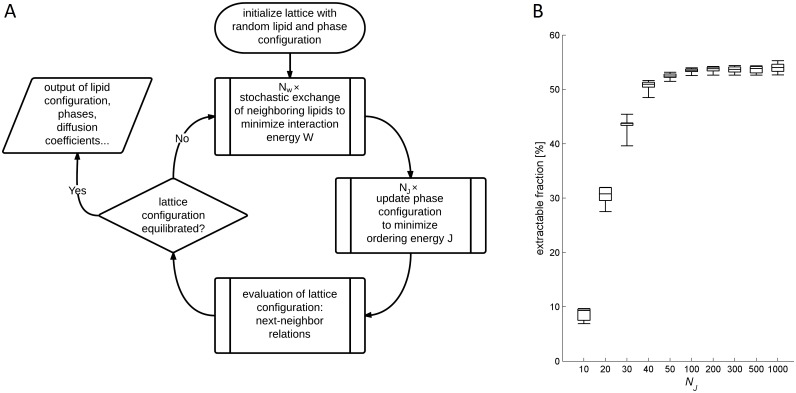
Algorithm scheme and influence of algorithm parameters. (A) Flow chart illustrating the 2-step minimization procedure applied to simulate the lattice dynamics and to calculate the ordering states of the lipids. (B) Box-plot depicting the influence of the control parameters *N_J_* (counting the number of updates of the ordering state carried out per lipid to minimize the ordering energy *J*) and *N_W_* (counting the number of pairwise lipid switches per lattice site in the minimization of the interaction energy *W*) on the phase separation of the model membrane. The parameter *N_J_* adopted different values between 10 and 1000 represented by the different boxes. For each value of *N_J_* the parameter *N_W_* adopted the values 1, 5, 10, 20, 50 and 100. For all combinations of *N_J_* and *N_W_* the percentage of the Ld phase (= extractable membrane fraction) was computed. The edges of the boxes indicate the lower and upper quartile, the horizontal bar inside indicates the median and the whiskers indicate the full range of the calculated values at fixed *N_J_* and *N_W_* varied.

Adopting the basic concept of multi-scale stochastic simulation of systems comprising a fast-equilibrium subsystem [30, 31] we split the simulation into two alternating steps: (1) A dynamic simulation governed by the interaction energy *W* and carried out over a critical time which is determined by the condition that each lipid has to change its position on the lattice *N_W_* times on average. During this dynamic simulation, the ordering states of the lattice sites are not changed, i.e. the ordering state is a fixed property of the lattice site and not of the lipid just occupying the site. The dynamic simulation step is followed by (2) the minimization of the total ordering energy *J*. This minimization step is carried out by the Metropolis algorithm [32] whereby the ordering states of all lattice sites are *N_J_* times updated. A change of the ordering state occurs with probability

$$P(\text{lo}↔\text{ho})=\{\begin{array}{c} 1\\ \exp(-\beta \Delta J) \end{array}\begin{array}{c} \text{if}\Delta J\leq 0\\ \text{if}\Delta J>0 \end{array}$$

depending on the difference Δ*J* between new and old ordering energy. The updated ordering states of the lipids are assigned to the harboring lattice sites and the simulation is continued with step (1) as depicted in Fig. 1A.

The whole simulation is either stopped at a fixed time point (this termination of the simulation was applied in the simulations of bile formation) or if the statistical properties of the lattice defined through frequency of lipid-lipid contacts, the relative share of lipids in lo states and ho states and the numerical value of the diffusion coefficient do not change over a sufficiently long time interval (this termination of the simulation was applied in the parameterization of the model based on experimental data with ternary lipid mixtures).

To ensure independence of the simulation results from the combination of the two linked optimization algorithms (see flowchart in Fig. 1A) the number of spin updates *N_J_* has to be high enough to minimize *J*. Likewise the number of lipid switches *N_W_* should be sufficiently low to ensure that the spin updates occur frequently enough. On the other hand it is desirable to keep *N_J_* low and *N_W_* high to minimize the computational effort. To find optimal values satisfying both requirements the control parameters *N_J_* and *N_W_* were varied and the dependence of the statistical properties of the model membranes was monitored. Fig. 1B shows an example demonstrating how the statistical properties of the simulated model configurations are influenced by the control parameters *N_J_* and *N_W_*. According to these results, we put *N_J_* = 100 and *N_W_* = 10 in the stochastic simulations.

The next step was to determine the interaction energies and the spin energies used in the model. With the three membrane lipids CH, PC and SM the symmetric matrices **w**_lo_ and **w**_ho_ of the interaction energies are 3×3 matrices comprising six independent unknown parameters *w*_σ_(CH, CH), *w*_σ_(CH, PC), *w*_σ_(CH, SM), *w*_σ_(PC, PC), *w*_σ_(PC, SM), and *w*_σ_(SM, SM), with σ = lo, ho state.

The matrix **j** of the ordering energies is a 6×6 matrix comprising 36 elements. The entries in the ordering matrix **j** describe the ordering energy of a given lipid (CH in the first column, PC in the second column, SM in the third column) with a neighboring lipid being in a given ordering state *σ*. The ordering state of the lipid itself is not of importance since in the used metropolis algorithm only energy differences are of importance, i.e. the calculation of Δ*J* leads to the same result if one puts $j({\text{X}}_{i}^{\text{lo}},{\text{X}}_{k}^{\text{lo}})=j({\text{X}}_{i}^{\text{lo}},{\text{X}}_{k}^{\text{ho}})$ and $j({\text{X}}_{i}^{\text{ho}},{\text{X}}_{k}^{\text{lo}})=j({\text{X}}_{i}^{\text{ho}},{\text{X}}_{k}^{\text{ho}})$. Therefore the dimension of the matrix is reduced to 3×6. The difference in the ordering energies for a given lipid with a neighboring lipid in either phase determines the tendency of the lipid to adopt either ordering state, with the lower value representing the favored and the higher value representing the disfavored ordering state. These pairs of values are block by block symmetric in the matrix which further reduced the free parameters from 18 to 6.

Thus, in total our model comprises 2·6+6 = 18 parameters with unknown numerical values.

We calibrated the interaction between the different lipids in different ordering states such that the diffusion coefficients would match those from experiment for the reported phases.

Our model allows to track the stochastic movement of individual lipids along with their ordering state and thus to determine mean squared displacements (MSDs) for the different phases. MSDs were calculated by first letting the simulation run until the resulting membrane configuration had reached an equilibrium state. At this point, we tagged the position of all *N* phospholipids *i*_0_ and let the simulation continue for a certain number of steps *z*. Next we calculated the individual displacement after the *z* steps for each lipid:

$$\Delta x(z,X_{i},\sigma_{i})=\left|i_{z}-i_{0}\right|$$

For the calculation of the displacements of the different lipid species X in the different phases only those random walks were taken into account for which the tracked lipid had not passed a phase border, i.e. had not changed its ordering state *σ_i_*.

Given the displacements Δ*x* and choosing an arbitrary timescale *t* corresponding to the *z* steps the simulated diffusion coefficient $D_{m,\sigma }^{\text{sim}}$ for the phase *σ* of the *m*-th model membrane can be derived from the MSDs with the corresponding ordering state in the respective lipid composition:

$$\langle \Delta x{\left(t\right)}^{2}\rangle =4D_{m,\sigma }^{\text{sim}}t.$$

The MSDs are only determined up to an overall scaling factor *α*:

$$\alpha =\frac{\sum_{m}D_{m,\sigma }^{\exp}D_{m,\sigma }^{\text{sim}}}{\sum_{m}{\left(D_{m,\sigma }^{\exp}\right)}^{2}}.$$

Here the index *m* designates the different lipid compositions of the GUVs, $D_{m,\sigma }^{\exp}$ denotes the measured and $D_{m,\sigma }^{\text{sim}}$ the simulated diffusion coefficient for lipid composition *m*.

The numerical values of the unknown elements of the matrices **w**_lo_ and **w**_ho_ were estimated by minimizing the difference *ε* between model-based lateral lipid diffusion rates and experimental values determined in giant unilamellar vesicles (GUVs) containing CH, PC and SM in 25 different proportions [33]:

$$\varepsilon =\sum_{m=1}^{25}{\left(D_{m,\sigma }^{\exp}-\alpha D_{m,\sigma }^{\text{sim}}\right)}^{2}\to \text{ minimum}.$$

calculated for a lattice having the same lipid composition *m* as the GUVs. Simulated diffusion coefficients that differ from the experimental ones by a global constant factor can be transformed by rescaling of *α* or alternatively by a rescaling of the time *t*. Therefor *α* relates the time scale used in the model simulations to the time scale of the *in vitro* experiments. The same value of the scaling factor *α* was used for fitting the unknown elements of the two matrices **w**_lo_ and **w**_ho_.

To solve this minimization problem, the numerical values of the 12 unknown parameters were varied on a discrete hypercube under the additional constraint of reproducing known interactions between the different lipid species. The condition for mixing of lipid species X and Y can be formulated as 2*w*_σ_(X, Y) − *w*_σ_(X, X) − *w*_σ_(Y, Y) < 0 while the condition for de-mixing is 2*w*_σ_(X, Y) − *w*_σ_(X, X) − *w*_σ_(Y, Y) > 0 [34]. The only constraints applied were that the lipid species CH and SM would mix in the ho state [35]. We determined the points of this hypercube that fulfilled the condition and where *ε* attained its minimal value for the ho or the lo state respectively. Around these points we again varied the unknown parameters on a finer hypercube and determined *ε*. The procedure was repeated with successively decreasing sizes of hypercubes until no further significant reduction of *ε* was possible. This corresponds to a discretized version of a downhill simplex method. The calibration yielded the following numerical values for the lipid—lipid interactions

$$w_{\text{ho}}=\left(\begin{array}{ccc} w_{\text{ho}}(\text{CH,CH}) & w_{\text{ho}}(\text{CH,PC}) & w_{\text{ho}}(\text{CH,SM})\\ & w_{\text{ho}}(\text{PC,PC}) & w_{\text{ho}}(\text{PC,SM})\\ & & w_{\text{ho}}(\text{SM,SM}) \end{array}\right)=\left(\begin{array}{ccc} -\text{0}.\text{70} & +\text{0}.\text{55} & -\text{1}.\text{70}\\ & -\text{0}.\text{60} & +\text{0}.\text{45}\\ & & -\text{0}.\text{80} \end{array}\right)$$

in the high ordered state and

$$w_{\text{lo}}=\left(\begin{array}{ccc} w_{\text{lo}}(\text{CH,CH}) & w_{\text{lo}}(\text{CH,PC}) & w_{\text{lo}}(\text{CH,SM})\\ & w_{\text{lo}}(\text{PC,PC}) & w_{\text{lo}}(\text{PC,SM})\\ & & w_{\text{lo}}(\text{SM,SM}) \end{array}\right)=\left(\begin{array}{ccc} -\text{0}.\text{55} & -\text{0}.\text{23} & +0.\text{43}\\ & -\text{0}.\text{22} & -\text{0}.\text{23}\\ & & -\text{0}.\text{55} \end{array}\right)$$

in the low ordered state.

For the interpretation of the numerical values of the interaction matrices we apply them to the mixing and de-mixing properties of the previous section and compare the results to known membrane properties. In the ho state the interaction matrix features a strong tendency for CH and SM to mix and a strong tendency for PC to de-mix from CH and from SM. This is in agreement with works from Silvius [35], van Duyl [36] and Frazier [37].

In the lo state PC has only a weak tendency to de-mix from CH and from SM, which is in agreement with work from Silvius [35] and Tsamaloukas [38]. Contrary to the ho state CH and SM have a tendency to de-mix in the lo state. For this pairing no reliable data could be found since the two species occur in the ho state rather than in the lo state.

The unknown parameters of the ordering matrix **j** were chosen such that the occurrence of monophasic and biphasic lipid distributions matched those observed in the 25 different variants of GUVs. To this end we defined lipid distributions with more than 90% of all lipids resident in the ho state as *monophasic liquid-ordered* (Lo), with more than 90% of all lipids resident in the lo state as *monophasic liquid-disordered* (Ld) and with more than 10% of all lipids resident in both ordering states as *biphasic*. For the minimization of *J* the lattice was initialized with all lipids in lo state. A searching of the parameter space yielded parameter values

$$j=\left(\begin{array}{c} \begin{array}{c} \begin{array}{ccc} j({\text{CH}}_{\text{lo}}\text{,CH}) & j({\text{CH}}_{\text{lo}}\text{,PC}) & j({\text{CH}}_{\text{lo}}\text{,SM}) \end{array}\\ \begin{array}{ccc} j({\text{CH}}_{\text{ho}}\text{,CH}) & j({\text{CH}}_{\text{ho}}\text{,PC}) & j({\text{CH}}_{\text{ho}}\text{,SM}) \end{array} \end{array}\\ \begin{array}{c} \begin{array}{ccc} j({\text{PC}}_{\text{lo}}\text{,CH}) & j({\text{PC}}_{\text{lo}}\text{,PC}) & j({\text{PC}}_{\text{lo}}\text{,SM}) \end{array}\\ \begin{array}{ccc} j({\text{PC}}_{\text{ho}}\text{,CH}) & j({\text{PC}}_{\text{ho}}\text{,PC}) & j({\text{PC}}_{\text{ho}}\text{,SM}) \end{array} \end{array}\\ \begin{array}{c} \begin{array}{ccc} j({\text{SM}}_{\text{lo}}\text{,CH}) & j({\text{SM}}_{\text{lo}}\text{,PC}) & j({\text{SM}}_{\text{lo}}\text{,SM}) \end{array}\\ \begin{array}{ccc} j({\text{SM}}_{\text{ho}}\text{,CH}) & j({\text{SM}}_{\text{ho}}\text{,PC}) & j({\text{SM}}_{\text{ho}}\text{,SM}) \end{array} \end{array} \end{array}\right)=\left(\begin{array}{c} \begin{array}{c} \begin{array}{ccc} 0.50 & 0.90 & 0.50 \end{array}\\ \begin{array}{ccc} 1.00 & 0.50 & 1.90 \end{array} \end{array}\\ \begin{array}{c} \begin{array}{ccc} 0.90 & 0.90 & 0.90 \end{array}\\ \begin{array}{ccc} 0.50 & 0.50 & 0.50 \end{array} \end{array}\\ \begin{array}{c} \begin{array}{ccc} 0.50 & 0.90 & 0.50 \end{array}\\ \begin{array}{ccc} 1.90 & 0.50 & 0.55 \end{array} \end{array} \end{array}\right)$$

that allowed to match 21 phases of the 25 model membranes.

The values thus obtained are by no means unique but this is not to be expected since the data described by them are only semi-quantative and the relative size of the ordering pairs for a given lipid to adopt either state is more important than the precise values.

**Figure 2:**
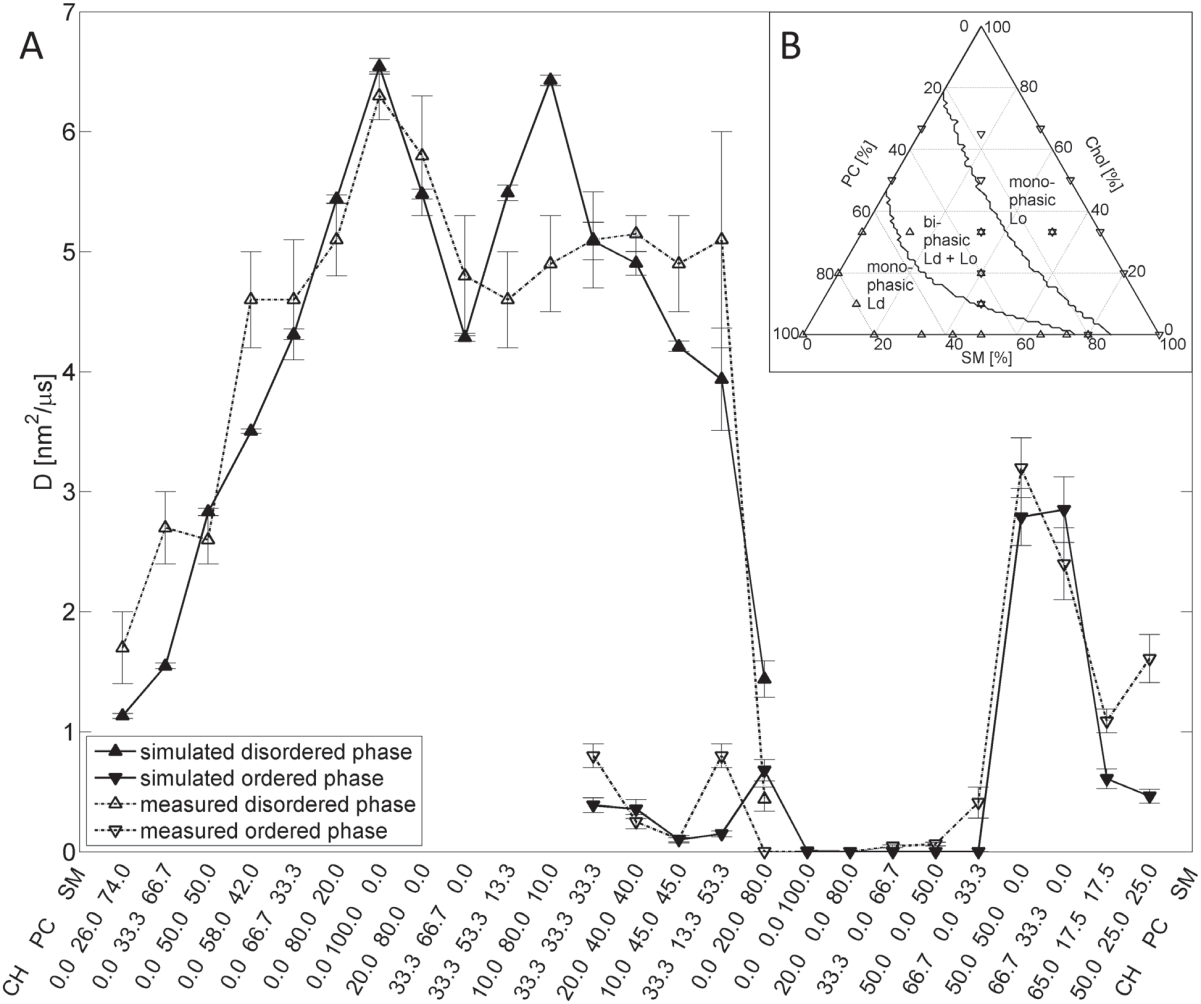
Lipid mobility and phase diagram. Simulation of lipid diffusion and lipid phases in GUV’s with varying lipid composition. (A) Simulated (filled markers) and measured (open markers) average diffusion coefficients in monolayers with 25 different mixtures of CH, PC and SM. Triangles facing up mark values of a liquid-disordered phase, triangles facing down of a liquid-ordered phase. Mixtures with both values show biphasic behavior, mixtures with either of both show respective monophasic behavior. The percentage of each lipid in the mixture is indicated at the bottom (CH—PC—SM). (B) Ternary phase diagram for mixtures of CH, PC and SM. The bold lines mark regions of lipid compositions for which the model predicts monophasic and biphasic behavior. In the monophasic Lo or Ld compositions more than 90% of the lipids are resident in the respective ordering state, whereas in the biphasic mixtures more than 10% of the lipids are resident in both ordering states thus favoring the formation of segregated microdomains. The triangles mark the measured lipid mixtures shown in (A) and indicate their measured phase state (up-facing triangle: Ld, down-facing triangle: Lo, star: biphasic) which not always matches our model prediction.

With these values the calculated diffusion coefficients and the occurrence of monophasic and biphasic lipid distributions of our model simulations were in agreement with experimental data obtained with GUVs [33] (see Fig. 2).

The calibration of the model with measured diffusion coefficients allows the definition of an absolute time scale and thus offers the possibility to use the model for real-time dynamic simulations of domain formation. We used the parameterized model to compute lipid distributions for the whole possible range of lipid compositions (Fig. 2B).

### Simulation of membrane self-organization and lipid secretion into the bile

We used the calibrated model to simulate the self-organization of nanodomains and the release of lipids from the outer leaflet of the canalicular hepatocyte membrane into the bile canaliculus. We presupposed a situation where the amount and composition of lipids in the outer leaflet is on the average kept constant (quasi steady state), i.e. the transport rate of lipids to the canalicular membrane equals the net transport rate from the inner to the outer leaflet and the release rate into the canalicular lumen.

As the life-time of nanodomain structures, i.e. the simulation time during which an initially random distribution of lipids self-organizes into a domain structure that is representative for the outer leaflet of the canalicular membrane, we chose *τ* = 0.1, 1 and 10 ms to cover a range around the estimated nanodomain life-time of ≈ 1 ms [39].

We tested two alternative mechanisms of lipid secretion: extraction of single lipids or extraction of lipid patches. An extractable lipid patch is defined as a hexagonal piece of membrane that is fully embedded in a Ld nanodomain. With this definition, extraction of single lipids is identical with extraction of patches with size of 1 lipid. We defined the extractable membrane fraction (EMF) as the fraction of the membrane that can be covered by extractable patches. The flow of lipids into the bile is determined by the detachment rate of patches. This rate depends on the concentration and chemical properties of the BS species present in the canalicular lumen and the size and number of patches present in the outer leaflet. Hence, at fixed concentration of BS, the lipid secretion rate (LSR) is up to a constant unknown factor proportional to the number of the extractable patches *n*_patch_(*r*) with radius *r* multiplied by their lipid content *n*_lipids_(*r*) and divided by the time span *τ* required for the formation of nanodomains:

$$\text{LSR}(r)=n_{\text{patch}}(r)n_{\text{lipids}}(r)/\tau .$$

The lipid composition of the bile is given by the average lipid composition of all extractable patches.

## Results

First, we carried out model simulations with a physiologically normal lipid composition of the canalicular membrane [8] with CH = 37.8%, PC = 46.5% and SM = 15.7%. The simulation was started with a fixed lipid composition but random distribution of lipids across the lattice with their ordering states calculated for the given random lipid distribution.

**Figure 3:**
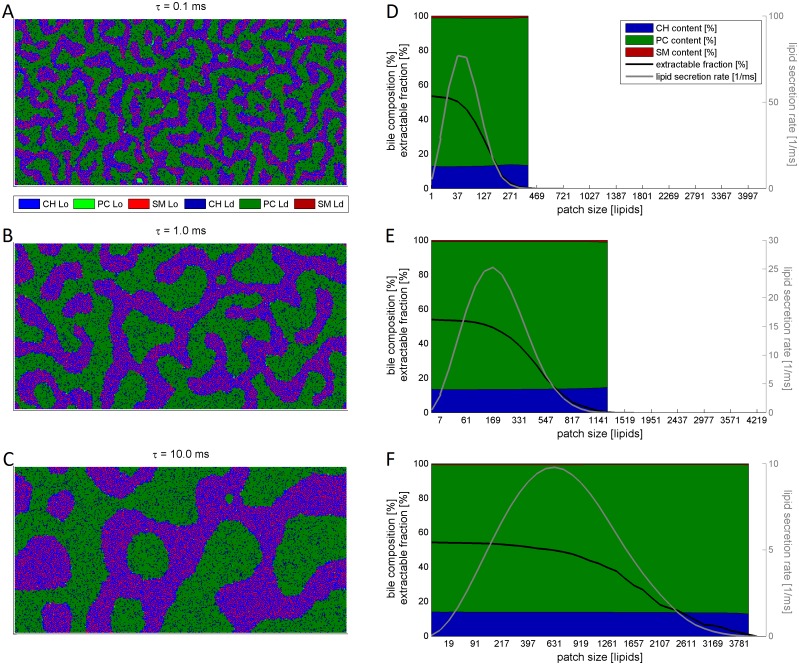
Membrane configurations and lipid extraction. Representative domain structures (A—C), EMF, LSR, and lipid composition (D—F) of BS-extractable membrane patches for three different life-times (=simulations times) of *τ* = 0.1, 1 and 10 ms. (A—C): At short life-times ((A), *τ* = 0.1 ms) maze-like structures are formed comprising closed Lo nanodomains (bright colors) enriched in CH (blue) and SM (red) which are imbedded into a sea of interconnected Ld nanodomains (dark colors) enriched in PC (green). With increasing life-times (B, C), the nanodomains merge with each other under formation of larger domains. (D—F) Shown as a function of the patch size are EMF (dashed curves), LSR (full curves) and bile composition (area in the background). While the absolute LSR varies, the lipid composition of BS extractable patches remains remarkably stable at varying patch sizes and fits well with observed lipid composition of the normal bile.

Model simulations persistently resulted in a bi-phasic quasi-stationary lipid distribution comprising Ld nanodomains rich in PC and Lo nanodomains enriched in CH and SM. Typical lipid patterns obtained for three different life-times are depicted in Fig. 3. With increasing life-time, the nanodomains tend to merge to form larger domains. Accordingly, the maximal size of patches that can be extracted from the Ld nanodomains increases as well. The predicted lipid composition of the patches and thus of the bile micelles was ≈ 13.5 CH, ≈ 85.5% PC and ≈ 1% SM in good agreement with reported experimental values of ≈ 15% CH, ≈ 85% PC and < 1% SM [40]. The lipid composition of patches was remarkably invariant against variations of the patch sizes and life-times (see Table 1).

**Table 1:** Lipid composition of the bile.

**Life-time**	**Patch size**	**CH**	**Phospholipids (= PC)**	**SM**
0.1 ms	r = 0, 1 lipid	13.0 ± 0.0%	85.7 ± 0.1%	1.3 ± 0.1%
	r = 3, 37 lipids	12.8 ± 0.2%	85.9 ± 0.2%	1.3 ± 0.1%
	r = 7, 169 lipids	13.3 ± 0.6%	85.6 ± 0.4%	1.4 ± 0.2%
	r = 9, 271 lipid	13.6 ± 1.4%	85.0 ± 1.4%	1.4 ± 0.1%
1 ms	r = 0, 1 lipid	13.6 ± 0.2%	85.5 ± 0.2%	0.9 ± 0.0%
	r = 3, 37 lipids	13.4 ± 0.2%	85.7 ± 0.2%	0.9 ± 0.1%
	r = 7, 169 lipids	13.5 ± 0.2%	85.6 ± 0.2%	0.9 ± 0.1%
	r = 14, 631 lipids	13.7 ± 0.6%	85.3 ± 0.5%	0.9 ± 0.3%
	r = 18, 1027 lipids	14.1 ± 0.5%	85.2 ± 0.8%	0.7 ± 0.4%
10 ms	r = 0, 1 lipid	14.2 ± 0.2%	85.1 ± 0.2%	0.7 ± 0.0%
	r = 3, 37 lipids	14.1 ± 0.2%	85.2 ± 0.2%	0.7 ± 0.1%
	r = 7, 169 lipids	14.1 ± 0.2%	85.2 ± 0.2%	0.7 ± 0.0%
	r = 14, 631 lipids	14.0 ± 0.1%	85.2 ± 0.1%	0.7 ± 0.0%
	r = 23, 1657 lipids	13.9 ± 0.4%	85.4 ± 0.3%	0.7 ± 0.1%
	r = 30, 2791 lipids	13.7 ± 0.6%	85.6 ± 0.6%	0.6 ± 0.1%
	Experiment [40]	15%	85%	< 1%

Predicted average lipid composition of circular membrane patches of different size in the liquid-disordered phase of the model membrane in comparison to experimentally observed values for different simulation times. The last patch size per simulation time is the largest patch that allowed lipid extraction from the membrane structure with the respective life-time.

As seen in Fig. 3D–F, the calculated LSRs are non-monotone with respect to the patch size. This is due to the fact that the number of lipids that are simultaneously extracted from the leaflet increases with the patch size whereas the number of patches fitting into Ld nanodomains decreases with increasing patch size. The largest LSR was attained for patches containing 37, 169 and 631 lipids for the three different simulation times *τ* = 0.1, 1 and 10 ms. This has to be compared with the size of sandwich-like micelles which primarily derive from mixed dispersions of egg PC and the BS deoxycholate [41] and quasi-elastic light scattering studies of native bile from the dog [42] consistently comprising about 100–400 lipids. Since this is close to the calculated average patch size at *τ* = 1 ms and as a life-time of 1 ms is also in good agreement with several measurements on nanodomains we choose *τ* = 1 ms as the average life-time in further simulations.

**Figure 4:**
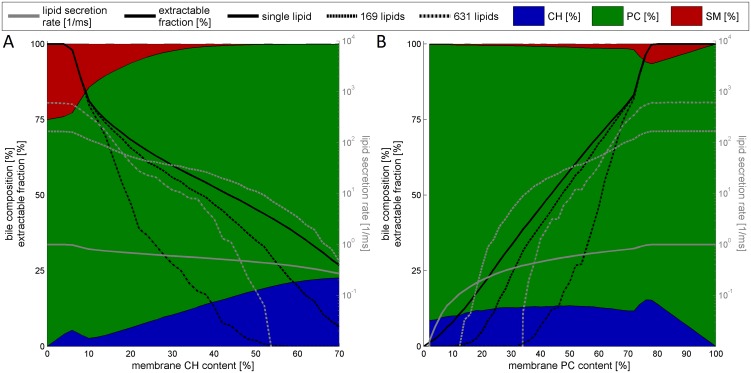
Influence of membrane lipid composition on lipid extraction. Simulated lipid extraction at varying CH and PC content of the exoplasmic leaflet of the apical membrane. Simulations refer to a life-time of 1 ms. Shown are the EMF (black curves) and the LSR (grey curves, logarithmic scale) as a function of CH content (A) or PC content (B) in the membrane for the extraction of single lipids and patches of 19, 169 and 631 lipids. The mean lipid composition of the soluble Ld nanodomains is depicted as blue (CH), green (PC) and red (SM) areas. (A) The CH content of the outer leaflet was varied from 0–70% at constant PC:SM ratio of 3:1. (B) The PC content of the outer leaflet was varied from 0–100% at a CH:SM ratio of 7:3.

The lipid composition of the canalicular membrane has been shown to strongly influence the relative share of membrane lipids in the bile [43]. Thus, we performed simulations where the relative fraction of either CH or PC was varied (see Fig. 4).

A decrease in the relative fraction of CH at otherwise constant PC:SM ratio increased the share of lipids in the Lo state and the average size of Ld nanodomains. As a consequence, the average size of membrane patches that can be solubilized from Ld nanodomains and the LSR increased with decreasing CH content of the external leaflet. At a low fraction of CH = 18% the model simulations predict a steep increase of the average patch size to about the 10-fold of the reference state. Such an increase of the BS-solubilizable membrane area should result in a BS-induced rupture of the membrane. Increasing the CH content of the exoplasmic leaflet promoted the transition from the Ld to the Lo phase and thus reduced the LSR. On the other hand, the CH content of the extractable membrane patches became successively larger. The net effect of these two opposing tendencies was an increase of the extractable fraction of CH up to a critical CH content which amounts to about 36% for extraction of patches with sizes of 100–400 lipids and to 56% for the mechanism of single-lipid extraction.

In a further series of simulations we studied the impact of varying concentrations of PC on the lipid flow. With decreasing PC content at constant CH:SM ratio the simulations revealed a non-linear decline of the average size of extractable patches resulting in reduced LSRs of all lipid species. The composition of the patches remained almost constant although there was a slight shift towards a higher share of CH and a lower share of PC.

Intriguingly, the lower limit of the membranous PC content below which the flow of PC into the bile practically ceased depended strongly on the size of membrane patches supposed to carry the lipid flow into the bile. For example, lipid extraction stopped at a lowered membranous PC content of 32% for patches with a size of 631 lipids, at 10% for 169 lipid patches while the extraction of single lipids did not stop until a PC content of 0%. Studies on mice with a homozygous knock-out of the PC transporter mdr2 found a complete abolishment of PC release into the bile [44]. Compared with our model simulation this finding again suggests lipid secretion to precede via extraction of patches rather than of single lipids. Increasing the PC content resulted in an increased EMF and strongly increased LSRs.

## Discussion

In this work we developed a kinetic model of lateral lipid diffusion and phase separation in monolayers composed of the three membrane lipids CH, DOPC and SM assumed to contain saturated fatty acid tails, even though in biological membranes the diversity of phospholipids is higher than in our three component system (e.g. phosphatidylserine, phosphoinositol, phosphatidylethanolamine) and the fatty acid tail composition is much more complex (hybrid, multiple unsaturated, different chain length). However studies by Marsh and Konyakhina show that the phase distribution between PC with one or two unsaturated fatty acid tails is rather similar [45–47] and PC is the dominant phospholipid species [8, 48]. Naturally mathematical modelling relies on justified simplification of real biological structures. In our model the influence of membrane proteins was neglected and DOPC is used in the model as a substitute for a number of different phospholipids (e.g. PE, PS) with the hybrid lipid 1-saturated, 2-unsaturated-phosphatidylcholine being the most abundant phospholipid. The differences between phase diagrams for DOPC/SM/CH and POPC/SM/CH are not necessarily higher than between different experiments of same the same system [49, 50]. In the end justification comes from the agreement between experiments and theory.

Therefore to keep the parameter space small and to allow for the comparison with experiment, the model was calibrated based on data from in vitro experiments with GUVs. We then used the model to study the dynamics of domain formation in the exoplasmic leaflet of the canalicular membrane of hepatocytes. The central goal of these simulations was to explore how the lipid composition of the leaflet influences the relative size of Ld and Lo nanodomains and thus the biliary phospholipid composition and the likelihood to solubilize membrane segments (patches) from the Ld nanodomains.

### Simulation of dynamic domain formation in the outer leaflet of the canalicular membrane

Importantly, at physiologically realistic lipid composition of the outer leaflet, our simulation consistently predicted the formation of two distinct types of nanodomains differing significantly in their lipid composition and phase behavior (Fig. 3). The Ld nanodomain was strongly enriched in PC while the Lo nanodomain was rich in CH and SM. These model-based findings are in good agreement with experiments clearly indicating a compartmentalization of lipids within the canalicular membrane [51]. Using either Triton X-100 or Lubrol WX as detergents, Slimane et al. [52] extracted two different membrane fractions. The Triton insoluble fraction was highly enriched in sphingolipids and CH [9]. The proteins mediating the trans-membrane lipid transport and the release of BS into the canalicular lumen (BS export pump, multidrug resistance protein 2, multidrug resistance associated protein 2, Abcg5) were found to be predominantly located in the Triton X-100 soluble fraction [51].

Experiments with model membranes devoid of proteins have provided evidence for the spontaneous formation of nano-scale lipid domains [19]. Whether spontaneous lipid de-mixing is also the primary mechanism for domain formation in biological membranes is a matter on ongoing debate (reviewed, for example, in [53]) as lipid—protein interactions may contribute not only to the stabilization of such domains but may even induce their formation. The fact that our lipid-based model of nanodomain formation in the canalicular membrane of hepatocytes indeed recapitulates a number of experimental findings on the size and lipid composition of bile micelles lends support to the view that spontaneous formation of pure Lo and Ld lipid domains without further assistance of membrane proteins is sufficient to enable the extraction of tiny membrane fragments from Ld domains in the presence of solubilizing agents. Whereas Ld domains are disrupted by the process of bile micelle formation and thus have to be permanently recreated, it is likely that the Lo lipid domains once formed are stabilized by the insertion of membrane proteins involved in the active transport for the various bile components and asymmetric distribution of lipids between the internal and external leaflet.

Intriguingly, Ld nanodomains obtained in our simulations contained PC, SM and CH in relative fractions that perfectly matched their relative abundance in the bile. This finding suggests that the three major lipids of the bile are not independently solubilized from the membrane but secreted in a concerted manner just in proportions present in the Ld nanodomains.

In our simulations the size of nanodomains increased with increasing life-times in a sub-linear fashion. Spectroscopic measurements suggest the life-times of nanodomains in biological membranes to be not longer than a few milliseconds. For this time window our simulations predict a maximal size of Ld nanodomains of only a few hundred lipids.

### Simulation of lipid extraction as exo-vesiculation of membrane patches into mixed micelles

Our lattice model allows calculating the size and geometry of nanodomains which represent important factors determining the rate with which membrane patches can be solubilized and extracted from the external leaflet. These simulations suggest that the LSR has a maximum for critical patch sizes of 100–400 lipids. Remarkably, the predicted optimal size of lipid patches is in good agreement with the size of micelles that are usually extracted from artificial membranes [28, 48]. Of note, even the largest patches that in our simulations were found to be extractable in a time window of a few microseconds were at least one order of magnitude smaller than bile vesicles observed by means of ultra-rapid cryofixation [7]. We suppose that these vesicles may derive from a fusion and rearrangement of smaller nano-micelles as those suggested by our simulations (micelle-to-vesicle transition, [54]). Such a mechanism would also better explain the formation of bilayered vesicles by pinch-off from a monolayer. The fact that some of the larger bile vesicles were found in direct contact with the canalicular membrane does not necessarily imply that they have originated from exocytosis, as suggested in [7]. Rather, they may represent vesicles that after their formation from micelles back-fuse with the canalicular membrane to be endocytosed [55, 56].

### Simulation of lipid extraction from membranes with altered phospholipid composition

Since altered phospholipid composition of the canalicular membrane can result in impaired bile formation and cellular damage we carried model simulations where the CH and PC content of the canalicular membrane was varied over a wide range (Fig. 4). A reduced PC content of the outer leaflet of the canalicular membrane can result from impaired mdr2 activity. mdr2 selectively transports PC to the outer membrane depending on BS concentration. Experimental studies on changes of bile flow and bile lipid composition in mice being homozygous for a disruption of the MDR2 gene, the analog of the human MDR3 [57, 58] revealed an only moderate decline of the PC flow into the bile in the heterozygous animals whereas in the homozygous mice the flow of PC and CH was almost completely abolished (lower than 5% of the normal). In our simulations a PC content of less than 30% completely prevented the extraction of patches with a size of about 600 lipids. In contrast, extraction of single lipids is predicted to continue—albeit with decreasing activity—if the PC content goes to zero. Hence, these simulations lend further support to the existence of a patch-extraction mechanism of membrane lipids. Unfortunately the residual PC content of the canalicular membrane has not been determined in the mdr2 knockout experiments so that a comparison with the predicted threshold value of about 30% PC content is not possible. However, considering that PC is an indispensable phospholipid of the plasma membrane and that besides mdr2 other transporters of PC exist, for example, the relatively unspecific MDR1 encoded P-glycoprotein [59], a residual PC content of 30% is not unlikely.

Finally we examined the dependence of the biliary phospholipid composition on the CH content of the outer canalicular membrane. Experimental data implicate that the heterodimer of the two half-transporters ABCG5 and ABCG8 translocates CH to the outer leaflet of the canalicular membrane [60, 61]. Mice with knockout of both transporters (Abcg5+/g8+) displayed strongly reduced biliary CH excretion [62]. This is reproduced by our simulations. The fraction of biliary CH shows a strong correlation with the CH content of the membrane. On the other hand the solubilizable fraction of the canalicular membrane decreases with increasing CH concentration demonstrating the ordering and stabilizing effect of CH [43]. Sufficient CH is required for stability of the canalicular membrane and protects cells from BS induced damage.

**Figure 5:**
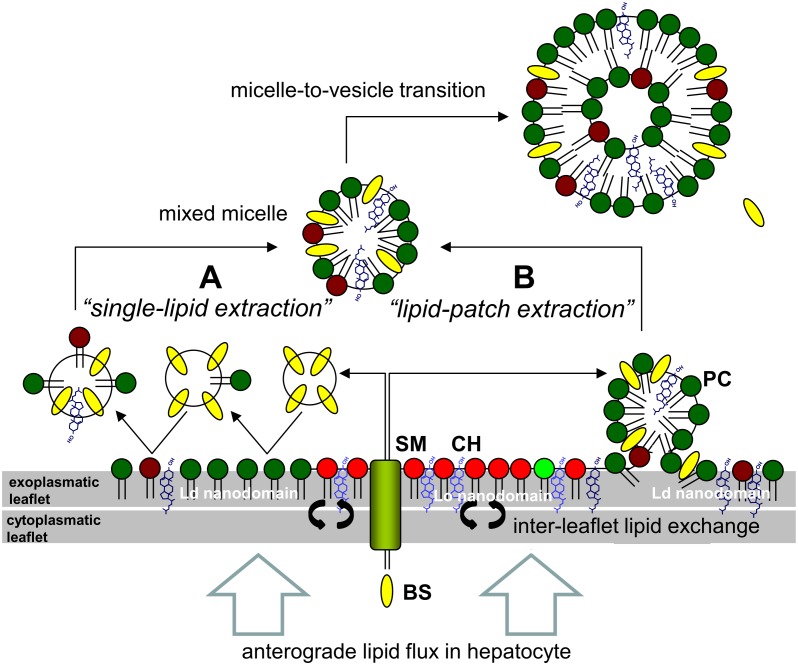
Illustration of the molecular mechanisms proposed for biliary lipid secretion. Lipids arrive at the apical membrane of the hepatocyte by various modes of transportation (vesicular, lipid-exchange proteins, lateral membrane transport). Bile salts (BS) are secreted into the canaliculus by the bile-salt export pump (BSEP). Inter-leaflet lipid exchange (indicated by the black double-arrows) by various ABC transporters and P-type ATPases (e.g. MDR1, MDR3, MRP1) establish an asymmetric lipid distribution between the inner and outer hemi-leaflet with CH, PC and SM enriched in the exoplasmic leaflet in a mixture which allows the spontaneous formation of Lo and Ld nanodomains. BS preferentially solubilize lipids of the Ld nanodomains. Mechanisms of lipid extraction currently discussed in the literature envisage (A) extraction of single membrane lipids (mostly PC) by BS micelles under formation of mixed micelles or (B) exo-vesiculation of membrane patches which form mixed micelles. Primary micelles may rapidly fuse to larger lipid vesicles (micelle-to-vesicle transition). The results of our simulation strongly favor the secretion mechanism B (lipid-patch extraction).

Taken together, our model-based calculations provide further evidence for the emergence of short-lived nanodomains in the external leaflet of the canalicular hepatocyte membrane. The best overall agreement between model simulations and experimental facts is achieved if we assume that the lipid transfer into the bile is mediated by small patches of 100–400 lipids which are extracted from the Ld nanodomains of the external leaflet by BS and which contain the main lipids CH, PC and SM in proportions as also found in the bile. Likely, the primary nano-micelles further maturate to larger lipid vesicles (see Fig. 5).
